# Relationship between low tube voltage (70 kV) and the iodine delivery rate (IDR) in CT angiography: An experimental *in-vivo* study

**DOI:** 10.1371/journal.pone.0173592

**Published:** 2017-03-20

**Authors:** Michael M. Lell, Ulrike Fleischmann, Hubertus Pietsch, Johannes G. Korporaal, Ulrike Haberland, Andreas H. Mahnken, Thomas G. Flohr, Michael Uder, Gregor Jost

**Affiliations:** 1 Department of Radiology and Nuclear Medicine, Paracelsus Medical University, Nuernberg, Germany; 2 Department of Radiology, Friedrich-Alexander University Erlangen, Erlangen, Germany; 3 MR and CT Contrast Media Research, Bayer Healthcare, Berlin, Germany; 4 Siemens Healthcare, Forchheim, Germany; 5 Department of Radiology, University of Marburg, Marburg, Germany; 6 Imaging Science Institute (ISI) Erlangen, Erlangen, Germany; Maastricht University Medical Centre, NETHERLANDS

## Abstract

**Objective:**

Very short acquisition times and the use of low-kV protocols in CTA demand modifications in the contrast media (CM) injection regimen. The aim of this study was to optimize the use of CM delivery parameters in thoraco-abdominal CTA in a porcine model.

**Materials and methods:**

Six pigs (55–68 kg) were examined with a dynamic CTA protocol (454 mm scan length, 2.5 s temporal resolution, 70 s total acquisition time). Four CM injection protocols were applied in a randomized order. 120 kV CTA protocol: (A) 300 mg iodine/kg bodyweight (bw), IDR = 1.5 g/s (flow = 5 mL/s), injection time (t_i_) 12 s (60 kg bw). 70 kV CTA protocols: 150 mg iodine/kg bw: (B) IDR = 0.75 g/s (flow = 2.5 mL/s), t_i_ = 12 s (60 kg bw); (C) IDR = 1.5 g/s (flow = 5 mL/s), t_i_ = 12 s (60 kg bw); (D) IDR = 3.0 g/s (flow = 10 mL/s), t_i_ = 3 s (60 kg bw). The complete CM bolus shape was monitored by creating time attenuation curves (TAC) in different vascular territories. Based on the TAC, the time to peak (TTP) and the peak enhancement were determined. The diagnostic window (relative enhancement > 300 HU), was calculated and compared to visual inspection of the corresponding CTA data sets.

**Results:**

The average relative arterial peak enhancements after baseline correction were 358.6 HU (A), 356.6 HU (B), 464.0 HU (C), and 477.6 HU (D). The TTP decreased with increasing IDR and decreasing t_i_, protocols A and B did not differ significantly (systemic arteries, p = 0.843; pulmonary arteries, p = 0.183). The delay time for bolus tracking (trigger level 100 HU; target enhancement 300 HU) for single-phase CTA was comparable for protocol A and B (3.9, 4.3 s) and C and D (2.4, 2.0 s). The scan window time frame was comparable for the different protocols by visual inspection of the different CTA data sets and by analyzing the TAC.

**Conclusions:**

All protocols provided sufficient arterial enhancement. The use of a 70 kV CTA protocol is recommended because of a 50% reduction of total CM volume and a 50% reduced flow rate while maintaining the bolus profile. In contrast to pulmonary arterial enhancement, the systemic arterial enhancement improved only slightly increasing the IDR from 1.5 g/s to 3 g/s because of bolus dispersion of the very short bolus (3s) in the lungs.

## Introduction

State-of-the-art multidetector-row computed tomography (MDCT) with large detector panels (up to 16 cm in the z-axis) and dual source technology with high-pitch scan modes have dramatically shortened data acquisition times. Computed tomography angiography (CTA) of single organs can be performed in a fraction of a second for example in coronary CTA [1]. Scanning at lower tube voltages (low-kV) increases the attenuation values of iodine because the resulting photon energy is closer to the iodine K-edge of 33 keV, resulting in higher intravascular enhancement. In CTA, this can be used to reduce the total contrast media (CM) dose while maintaining image quality or to reduce the radiation dose at constant image quality [2–4].

Previously, the use of low-kV protocols in fast thoraco-abdominal CTA was restricted to children and patients with a low body mass index because of insufficient tube output capacity [5], but the latest CT systems mitigate this problem with more powerful x-ray tubes [2, 6–9]. When the scan time of a body CTA is on the order of 5 s or less, the traditional CM injection protocols need to be reconsidered and short and compact injection protocols should be preferred to those protocols that are optimized to provide a long and stable plateau phase of enhancement [10]. High peak enhancement with low CM volume can be achieved with monophasic injection protocols and a high iodine delivery rate (IDR) [11, 12], and it has been demonstrated, that even very high flow rates can safely injected with special i.v. lines [13].

The aim of our study was to optimize CM delivery parameters, particularly the injection duration and iodine delivery rate (IDR), using systematic evaluation of the time attenuation curves (TAC) in low-kV thoraco-abdominal CTA acquired with dynamic CTA that covers the thoraco-abdominal aorta with high temporal resolution in a porcine model.

## Materials and methods

### Anesthesia and animal preparation

Six female pigs with a mean body weight of 58.0 kg (range: 55–68 kg) were included in this study after approval from the state committee on animal affairs (LaAGeSo IC 113-A-0137/91) and the institutional review board of the University Erlangen. Anesthesia was induced by an intramuscular injection of 30 mg/kg body weight ketamine (Ketavet 10%; Pharmacia, Karlsruhe, Germany), 2 mg/kg azaperonum (Stresnil; Janssen-Cilag, Neuss, Germany), and 1 mg of atropine (Atropinum SULF 0.5 mg; Eifelfango, Bad Neuennahr-Ahrweiler, Germany). An 18G venous access was established bilaterally in an ear vein. After intravenous application of 1.4 mg/kg propofol (Propofol-ratiopharm; Ratiopharm, Ulm, Germany), the animals were orally intubated (Roesch tube 6.0; Teleflex Medical, Kernen, Germany) and mechanically ventilated with an oxygen-air-mixture using an anesthesia workstation (Fabius CE; Draeger Medical, Luebeck, Germany). Anesthesia was continued during the entire experiment by an intravenous propofol infusion of 20 mg/kg/h. Oxygenation, and the heart rate were continuously monitored (Infinity Delta; Draeger Medical, Luebeck, Germany).

### Dynamic computed tomography angiography

The animals were positioned in the prone position on the table of a 192-slice DSCT scanner (Somatom Force; Siemens Healthcare GmbH, Forchheim, Germany). CM (Ultravist 300; Bayer Vital, Leverkusen, Germany) was delivered with a power injector (Medrad Stellant; Bayer HealthCare, Leverkusen, Germany) via the 18 G i.v. line in the ear vein contralateral to the anesthetic drug delivery. All scans were performed during an end-expiratory breath hold. A dynamic scan mode with a scan range of 454 mm was used to obtain continuous data over a period of 70 s with a temporal resolution of 2.5 s, resulting in 28 CTA data stacks per scan. Dynamic CTA was performed with tube voltages of 120 kV and 50 mAs (reference) and 70 kV and 120 mAs, respectively. The CTDI_vol_ values were 102.4 mGy (120 kV) and 43.1 mGy (70 kV). Data acquisition and CM injection were started simultaneously. Because the enhancement values depend on the tube voltage, 300 mg iodine per kg body weight (mg I/kg) was injected for the standard protocol (120 kV), and 150 mg I/kg was injected for the 70 kV protocol. CM was injected near body temperature (38°C) and was followed by 25 mL of saline at the same injection speed. Four different CM-injection protocols (Table 1) were applied in random order in all animals, with a resting time of at least 40 min between two consecutive injections. The injection parameters (peak pressure and peak flow of CM, peak pressure and peak flow of saline) were monitored using the Certegra informatics platform (Bayer HealthCare, Leverkusen, Germany). Finally, the animals were euthanized with 50 mg/kg pentobarbital (Eutha 77; Essex Animal Health, Friesoythe, Germany).

**Table 1:** Contrast media protocols.

Protocol	tube voltage	iodine dose	IDR	flow rate	injection time (60 kg bw)
A	120 kV	300 mg I/kg	1.5 g/s	5 mL/s	12 s
B	70 kV	150 mg I/kg	0.75 g/s	2.5 mL/s	12 s
C	70 kV	150 mg I/kg	1.5 g/s	5 mL/s	6 s
D	70 kV	150 mg I/kg	3 g/s	10 mL/s	3 s

mg I/kg: milligram iodine per kilogram bodyweight; IDR: iodine delivery rate; kg bw: kilogram bodyweight

### Image reconstruction, post-processing and data analysis

Twenty-eight CTA data stacks were reconstructed from each raw-data set with the following reconstruction parameters: 3 mm slice thickness, 2 mm slice increment, soft tissue reconstruction kernel (Br36f), field-of-view of 320 × 320 mm^2^, and a reconstruction matrix of 512^2^. The dynamic data were analyzed on a post-processing platform (Syngo.via, VA 30, Siemens Healthcare GmbH, Erlangen, Germany). Motion correction was performed, registering data onto a reference volume using non- rigid registration to remove residual misalignment. TAC were created by placing ROIs into the following vessel segments: right and left upper- and lower-lobe pulmonary arteries, ascending aorta, brachiocephalic trunk, descending aorta at the level of the mitral valve, descending aorta at the level of the diaphragm (aortic hiatus), abdominal aorta at the level of the celiac trunk, and left and right renal artery. ROI were drawn by a board certified radiologist and double-checked by another board certified radiologist (both >15 years of experience in CTA) as large as possible, avoiding vessel walls. To correct for residual enhancement from previous scans, all measurements were baseline-corrected (HU–HU_t = 0_). The TAC were exported to a text file and transferred into Excel (Excel 2013; Microsoft Corp, Redmond, WA, USA).

To test the homogeneity of aortic enhancement with the different protocols, a centerline along the aorta was created and HU measurements every 2 mm along this centerline were taken.

The diagnostic window, i.e. time period of the bolus in which the baseline corrected attenuation exceeded 300 HU (t_300HU_) was extracted from the TAC. The TAC of the descending aorta at the level of the aortic hiatus were exported to an external workstation and a gamma-variate fit was applied to the measurement points using a nonlinear least square fit in order to extrapolate data with a temporal resolution of 0.1 s (MATLAB 7.1, The MathWorks, Natick, MA, USA) [14]. Based on the fitted curve, the optimal delay time for bolus tracking for a single-phase CTA was calculated. This delay was defined as the time between trigger level (arbitrarily set at 100 HU) and baseline corrected (relative) target enhancement of 300 HU was reached.

For qualitative analysis all individual CTA data sets were independently reviewed by two observers (1 and >15 years’ experience in CTA; both blinded to all identifying information) to depict the best angiographic phases that demonstrated high arterial contrast in small arteries without interfering venous enhancement. Cases of disagreement were solved in a consensus reading that joined a third reader (>15 years’ experience in CTA). We did not use a scoring system but used a dichotomous judgment (insufficient enhancement or venous overlay–sufficient arterial enhancement without or with minimal venous overlay).

### Statistical analysis

Continuous data are expressed as the arithmetic mean ± standard deviation (SD). All HU values are baseline corrected unless otherwise specified. The intra-individual comparisons of the CM injection protocols (A-D) for peak enhancement and time to peak (TTP = start of injection to peak enhancement) were calculated using two-way ANOVA followed by a multiple comparison post-hoc Tukey test. One-way ANOVA was used for the heart rate injection pressure, FW300 and bolus tracking delay time and was again followed by a multiple comparison post-hoc Tukey test. The significance level was set to p<0.05. All calculations were performed using GraphPad Prism (GraphPad Software Inc., La Jolla, CA, USA).

## Results

All examinations could be performed successfully without complications in all animals. The heart rate during CM injection did not differ significantly among the different injection protocols (p = 0.395), and the mean heart rate was 74.7±15.9 (protocol A), 79.0± 20.3 (protocol B), 77.6± 15.9 (protocol C), and 80.9± 13.6 (protocol D) beats per minute.

The peak CM injection pressure differed significantly (p<0.0001), was dependent on the injection speed (flow rate) and ranged from 37.0 to 305.0 psi. Similar mean peak injection pressures without significant differences were measured for protocols A (115.3±9.8 psi) and C (114.5±15.7 psi), both delivering CM at a flow rate of 5 mL/s (IDR 1.5 g/s). The lowest mean peak injection pressure was measured for protocol B (43.7±4.5 psi), delivering CM at a flow rate of 2.5 mL/s (IDR 0.75 g/s); the highest mean peak injection pressure was measured for protocol D (277.3±35.5 psi; IDR 3 g/s). The pressure limit of the injector was not reached in any of the animals, no local CM extravasation occurred.

**Fig 1:**
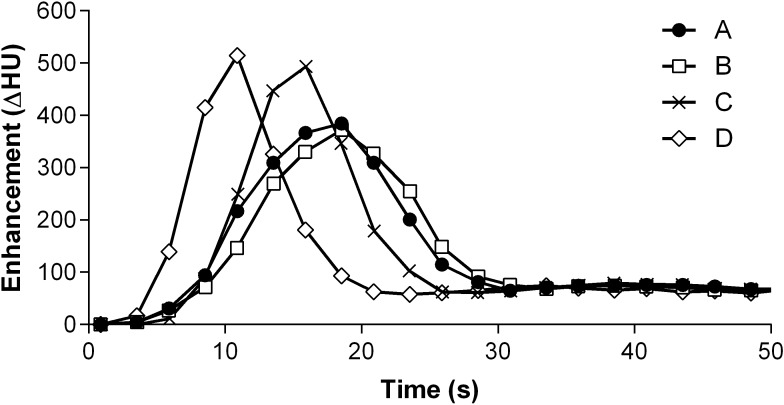
**Group averaged TAC’s adjusted to identical peak time for protocols A, B, C and D measured in the descending aorta**.

**Fig 2:**
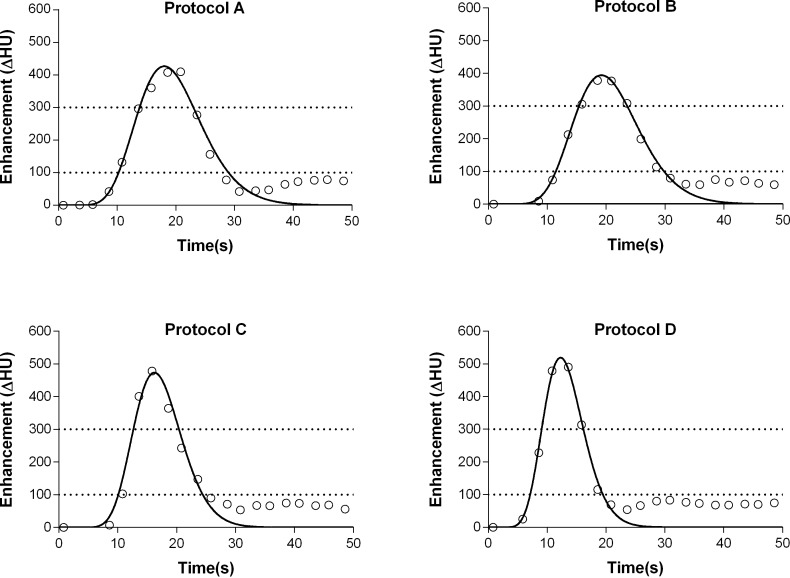
**Fitted time attenuation curves of the aorta from a representative animal for protocols A, B, C and D.** The dotted line at 100 HU represents the bolus tracking threshold level; the dotted line at 300 HU represents the diagnostic target attenuation. The diagnostic window (enhancement > 300 HU) is comparable for all protocols (7–9 s), and the bolus tracking delay time is shorter for protocols C and D than that for protocols A and B.

**Fig 3:**
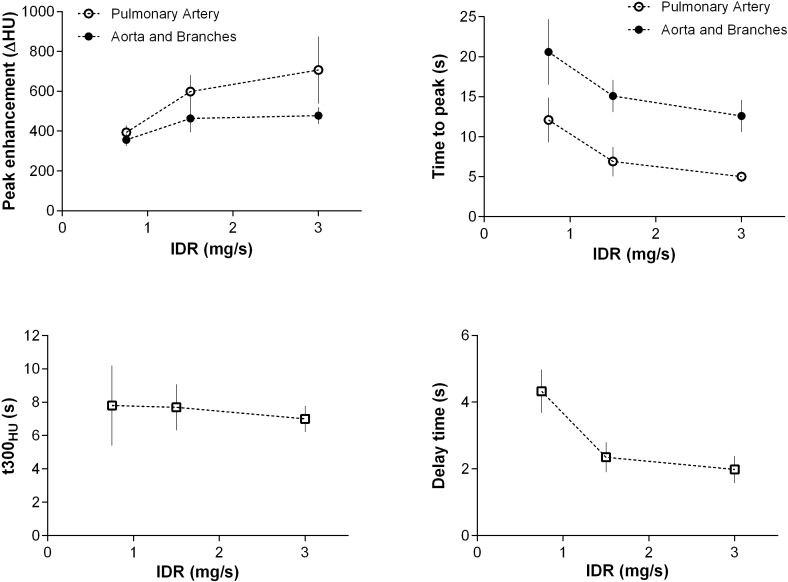
Effect of the IDR on peak enhancement and time to peak in 70 kV CTA. Correlation of peak enhancement and time to peak of the pulmonary arteries and aorta (for the different IDR of the CM injection protocols. The diagnostic window (t300 HU) and the bolus tracking delay time are given for the aorta and its main branches.

Sufficient arterial peak enhancement (baseline corrected arterial enhancement > 300 HU) was achieved with all protocols (Table 2 and Figs 1–3). The average peak enhancement values within the pulmonary arteries were 435.5±42.7 HU (protocol A), 393.4±35.8 HU (protocol B), 598.5±83.8 HU (protocol C), and 707.2±168.4 HU (protocol D); after adjusting for multiple comparisons, all differences were statistically significant (p< 0.001) except for protocol A versus B (p = 0.372). TTP correlated with the injection time and was shortest with protocol D (5.0±0.66 s) and longest for protocol B (12.1±2.8 s) and A (10.9±2.5 s). Again, all differences except that for A versus B (p = 0.183) were significant (p<0.01).

**Table 2:** Peak arterial enhancement (HU, mean ± standard deviation) for injection protocol A-D.

	A			B			C			D		
right superior PA	430.7	±	35.5	396.1	±	30.1	596.2	±	105.5	739.2	±	135.9
right inferior PA	422.4	±	24.1	394.9	±	29.4	603.7	±	90.5	743.9	±	149.2
left superior PA	447.1	±	65.6	389.4	±	38.0	591.2	±	74.7	674.8	±	205.1
left inferior PA	441.3	±	76.8	393.3	±	55.5	602.8	±	84.8	670.9	±	205.8
ascending aorta	341.0	±	33.7	350.4	±	29.3	458.5	±	71.7	497.1	±	38.2
brachiocephalic trunk	371.3	±	41.6	370.5	±	33.4	454.0	±	65.3	473.5	±	51.1
descending aorta (mv)	356.2	±	37.9	363.8	±	38.8	460.8	±	63.8	492.2	±	56.8
descending aorta (d)	392.0	±	40.5	372.6	±	30.8	493.5	±	66.6	525.6	±	53.0
abdominal aorta (ct)	380.5	±	36.7	360.2	±	32.3	496.6	±	74.2	497.9	±	58.3
abdominal aorta (ra)	379.1	±	47.1	370.0	±	26.7	492.8	±	86.9	474.2	±	37.1
left renal artery	315.1	±	61.3	322.0	±	48.6	427.1	±	85.9	414.8	±	36.0
right renal artery	333.7	±	50.7	343.2	±	49.8	428.5	±	85.9	445.2	±	60.3

PA pulmonary artery; mv: level of mitral valve; d level of diaphragm (aortic hiatus); ct: level of celiac trunk; ra: level of renal arteries. All HU values are baseline corrected to avoid bias from residual intravasal CM from previous scans.

The average peak enhancement values within the aorta and branches were 358.6±33.7 HU (protocol A), 356.6.±31.6 HU (B), 464.0±70.3 HU (C), and 477.6±42.2 HU (D); after adjusting for multiple comparisons, differences were statistically significant (p< 0.001) except that for protocol A versus B (p = 0.998) and C versus D (p = 0.564). Data from all arterial segments are given in Table 2. TTP was shortest for protocol D (12.6±2.0 s) and longest for protocol B (20.6±4.1 s) and A (20.1±3.3 s) (Table 3). All differences except that for A versus B (p = 0.843) were significant (p<0.001; Table 4).

**Table 3:** Time-to-peak (s, mean ± standard deviation) for injection protocol A-D.

	A			B			C			D		
right superior PA	10.4	±	3.2	12.0	±	2.6	6.6	±	1.9	4.7	±	0.7
right inferior PA	10.4	±	3.2	11.2	±	3.7	6.8	±	2.3	4.7	±	0.7
left superior PA	11.5	±	2.0	13.1	±	2.8	7.0	±	2.2	5.3	±	0.8
left inferior PA	11.5	±	2.0	12.0	±	2.9	7.0	±	2.2	5.3	±	0.8
ascending aorta	19.4	±	2.8	19.7	±	4.0	13.8	±	1.9	12.4	±	2.4
brachiocephalic trunk	20.0	±	3.2	19.8	±	4.4	13.9	±	1.9	12.2	±	2.6
descending aorta (mv)	19.5	±	3.2	19.5	±	4.5	13.9	±	1.8	12.1	±	2.4
descending aorta (d)	19.6	±	3.4	20.1	±	4.0	16.0	±	2.2	12.6	±	2.0
abdominal aorta (ct)	19.7	±	3.7	21.1	±	4.3	15.8	±	2.3	12.8	±	1.9
abdominal aorta (ra)	19.9	±	3.8	21.2	±	4.1	15.7	±	2.4	12.9	±	1.9
left renal artery	21.3	±	4.6	21.5	±	3.9	16.0	±	2.6	12.9	±	1.9
right renal artery	21.6	±	4.8	21.5	±	4.0	15.7	±	2.4	13.0	±	1.9

mv: level of mitral valve; d level of diaphragm (aortic hiatus); ct: level of celiac trunk; ra: level of renal arteries

**Table 4:** Statistical evaluation of peak enhancement (Peak), time-to-peak (TTP), the diagnostic window for an enhancement > 300 HU (t_300HU_) and the bolus tracking delay time (Delay Time): p-values for multiple comparison among injection protocols A-D.

	Pulmonary arteries		Aorta and branches		Descending aorta*	
	Peak	TTP	Peak	TTP	t_300HU_	Delay time
A vs. B	0.3715	0.1826	0.9975	0.8426	0.9402	0.2973
A vs. C	< 0.0001	< 0.0001	< 0.0001	< 0.0001	0.9270	< 0.0001
A vs. D	< 0.0001	< 0.0001	< 0.0001	< 0.0001	0.3890	< 0.0001
B vs. C	< 0.0001	< 0.0001	< 0.0001	< 0.0001	>0.9999	< 0.0001
B vs. D	< 0.0001	< 0.0001	< 0.0001	< 0.0001	0.7126	< 0.0001
C vs. D	0.0005	0.0072	0.5643	0.0001	0.7374	0.3677

* central slice position within the dynamic scan range; 2.5 s between each data point.

The delay time for bolus tracking for single phase CTA was comparable for protocol A and B (3.9±0.5 s and 4.3±0.7 s) and C and D (2.4±0.4 and 2.0±0.4 s) (Fig 3).

The diagnostic window (t_300HU_) was similar for all protocols on average (8.2±2.3 s (A); 7.8±2.4 s (B); 7.7±1.4 s (C); 6.9±0.8 s (D); all p>0.05) (Fig 3).

Visual assessment regarding clear delineation of the arterial system without significant venous contamination revealed an average time frame of 5.8±2.0 s for protocol A, 5.8±2.0 s for protocol B, 5.0±0.1 s for protocol C and 5.8±1.3 s for protocol D. These time frames correlated well with the t_300HU,_ although they were shorter because of the short arterio-venous transit time in the kidneys. Within all other organs of the chest and abdomen, the time frames of excellent arterial visualization were similar to those of t_300HU_.

Baseline corrected enhancement levels > 400 HU were consistently achieved with protocols C and D only. The diagnostic window (t_400HU_), was 4.8±1.9 s (C) and 4.6±0.9 s (D).

**Fig 4:**
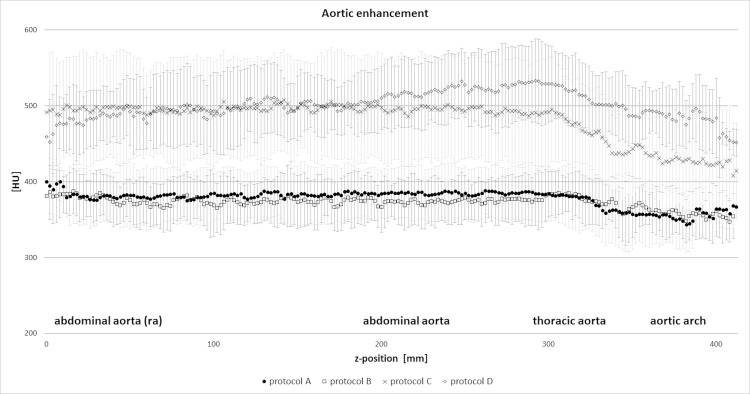
Relative HU-enhancement within the aorta for the different protocols at the optimal delay time. Measurements were taken along a centerline of the aorta. The markers indicate the mean, the error-bars the variation between the different animals for each protocol.

Enhancement values along the aorta were relatively homogeneous, especially for protocol A and B (Fig 4).

## Discussion

The aim of this study was to optimize CM delivery parameters in state-of-the-art CTA. We propose using a low-kV CTA to reduce the CM volume and injection speed. Similar enhancement of the arteries of the pulmonary and systemic circulation could be achieved with 50% of CM volume and 50% injection speed with 70 kV as compared to a 120 kV CTA. For dynamic or perfusion CT (PCT), very rapid injection of relatively small CM volumes and scanning at low kV has been recommended [12, 15–17]. The short and compact CM bolus is required to satisfy the assumptions of the perfusion models. CTA, either of single organs like coronary CTA or larger regions like the complete aorta, can be performed in a very short acquisition time (< 1s to approximately 5s) with modern CT systems [3, 18–20]. So traditional multiphasic injection protocols, developed to achieve stable, high arterial enhancement throughout the data acquisition period [21, 22] are no longer required and the absolute height of the TAC is the primary aim of protocol optimization. Thus, the requirements for CTA and PCT become very similar [23, 24].

We measured similar enhancement of the arteries of the pulmonary and systemic circulation and received similar respective TAC using a standard protocol (120 kV) and a 70 kV protocol when the injection time is kept constant and the IDR is adapted through lowering the injection speed (50%). The reduction of the flow rate from 5 mL/s to 2.5 mL/s resulted in a reduction of the injection pressure by more than 60%, which might add to patient comfort and allow the use of smaller caliber i.v. lines or the injection through port-catheter systems. The flow rate of 5 mL/s at 70 kV lead to higher peak enhancement, a similar injection pressure, a mean scan window t_300HU_ of approximately 8 s, and a reproducible elevated scan window at a level > 400 HU of t_400HU_ = 5 s. The visual evaluation of the data sets confirmed the results from the TAC analysis with shorter delay time at increasing IDR but similar scan window lengths for all tested injection protocols. For certain vascular territories, venous “contamination”, a too-high enhancement of the veins during arterial-phase imaging, can impair the diagnostic value of the CTA. This is why the visually determined optimal scan windows were shorter than the ones derived from the arterial TAC. A further increase in the flow rate to 10 mL/s resulted in a very short injection duration, with a slightly narrower mean scan window t_300HU_ of 7 s and t_400HU_ of approximately 5 s, with both differences being small and clinically non-relevant. The average pulmonary arterial enhancement increased significantly compared with a 5 mL/s injection, a finding that has been confirmed in a clinical study in patients with pulmonary embolism [25]. Although the injection pressure increased by a factor of 2.4, the average aortic enhancement was not significantly higher. This can be explained by the effect of dispersion of a very short bolus during the heart-lung passage, discouraging the use of such extreme flow rates in low CM-volume CTA of the systemic circulation [26, 27].

In clinical practice, factors other than maximal peak enhancement within the target vessel are worth considering. Injection pressure is related to patient comfort and safety, and the risk of automatic down regulation or cessation of the CM injection by the power-injector. Even with flow rates of 10 mL/s we did not reach the pressure limit using preheated contrast material (18 G i.v. line, 300 mg I/mL, 38° C). This can be different with conventional, small-caliber i.v. lines, a lower temperature of the CM, or higher concentrated CM [28]. Timing needs to be individualized, using either a test bolus or bolus tracking procedure [29, 30]. It has been demonstrated that the shape of the full bolus TAC can be reliably predicted from the test bolus TAC [31]. Bolus tracking is the alternative procedure that is frequently applied because of its ease of use. Usually, a short interscan delay (≥ 2 s) is required to move the table to the starting position and for the breathing command. The interscan delay did not interfere with the bolus tracking delay (2–4.3 s) and the scan window of t_300HU_ > 6.9 s and t_400HU_ > 4.8 s of any of the protocols. Because the scan time is very short, only a short cessation of breathing is required, that can be achieved during the interscan delay.

There are limitations to be addressed. All of the measurements were performed in young healthy pigs without cardiovascular disease. To adjust for physiologic changes during the study period, we randomized the order of the protocols. However, high-grade stenosis, occluded segments, or aneurysms that alter flow dynamics, cannot be simulated using our study setup. Because we performed dynamic scanning of the chest and abdomen over a 70 s period, equivalent CNR for the protocols were not intended because of tube limitations. For a single CTA, however it is certainly possible to achieve similar noise levels. It has been demonstrated previously, that 70 kVp CTA allows for significant radiation dose savings with comparable SNR and CNR [20], and iterative reconstruction techniques are commonly employed to further reduce image noise [6]. In contrast to dynamic CTA without table movement, the temporal resolution of dynamic spiral acquisition with continuous table movement back and forth is reduced and the time interval between two measurements at the same slice position is not identical except at the central z-position. However, dynamic spiral CTA allows a much larger z-coverage (45.4 cm in our study) than dynamic CTA without table movement (z-coverage up to 16 cm), and contrast enhancement in multiple locations or organs can be assessed.

## Conclusions

In this study, we conclude that arterial enhancement depends upon tube voltage and IDR. All tested protocols resulted in sufficient arterial and homogeneous enhancement (> 300 HU) throughout the aorta. 50% of CM could be saved using 70 kV and similar enhancement levels were achieved with a 50% lower injection rate. A scan window > 400 HU could be consistently achieved with 5 mL/s (IDR of 1.5 g/s) and higher at 70 kV, but injection of CM with 10 mL/s (IDR = 3 g/s, 3 s injection time) resulted in high pulmonary but only moderately increased systemic arterial enhancement levels due to bolus dispersion during the heart lung passage.
